# Supplementary material for: A fault-intrusion-tolerant system and deadline-aware algorithm for scheduling scientific workflow in the cloud
Source: PeerJ Comput Sci. 2021 Nov 2;7:e747. doi: 10.7717/peerj-cs.747 (PMC8576554; doi:10.7717/peerj-cs.747)
Supplement: Supplemental Information 6 [file peerj-cs-07-747-s006.zip › Myproject1.1/docs/org/workflowsim/clustering/balancing/methods/package-tree.html]

org.workflowsim.clustering.balancing.methods Class Hierarchy


JavaScript is disabled on your browser.


- Overview
- Package
- Class
- Use
- Tree
- Deprecated
- Index
- Help

- Prev
- Next

- Frames
- No Frames

- All Classes

# Hierarchy For Package org.workflowsim.clustering.balancing.methods

Package Hierarchies:

- All Packages

## Class Hierarchy

- java.lang.Object
  - org.workflowsim.clustering.balancing.methods.BalancingMethod
    - org.workflowsim.clustering.balancing.methods.ChildAwareHorizontalClustering
    - org.workflowsim.clustering.balancing.methods.HorizontalImpactBalancing
      - org.workflowsim.clustering.balancing.methods.HorizontalDistanceBalancing
    - org.workflowsim.clustering.balancing.methods.HorizontalRandomClustering
    - org.workflowsim.clustering.balancing.methods.HorizontalRuntimeBalancing
    - org.workflowsim.clustering.balancing.methods.VerticalBalancing

- Overview
- Package
- Class
- Use
- Tree
- Deprecated
- Index
- Help

- Prev
- Next

- Frames
- No Frames

- All Classes
